# Cause-specific child mortality performance and contributions to all-cause child mortality, and number of child lives saved during the Millennium Development Goals era: a country-level analysis

**DOI:** 10.1080/16549716.2018.1546095

**Published:** 2018-11-26

**Authors:** Yan Jin, Paul Mansiangi Mankadi, Jose Irineu Rigotti, Seungman Cha

**Affiliations:** a Department of Microbiology, Dongguk University College of Medicine, Gyeongju, Republic of Korea; b Environmental Health Department, School of Public Health, University of Kinshasa, Kinshasa, Democratic Republic of the Congo; c Department of Demography, Federal University of Minas Gerais, Belo Horizonte, Brazil; d Faculty of Infectious and Tropical Disease, London School of Hygiene & Tropical Medicine, London, UK; e Takemi Program in International Health, Global Health and Population Department, Harvard T.H. Chan School of Public Health, Boston, MA, USA

**Keywords:** Annual average reduction rate, cause-specific child mortality, Millennium Development Goal (MDG) era, cause-specific contribution to child mortality, number of child lives saved

## Abstract

**Background**: During the Millennium Development Goal (MDG) era, impressive reductions in the under-5 mortality rate (U5MR) have been observed, although the MDG 4 target was not met. So far, cause-specific progress in child mortality has been analyzed and discussed mainly at the global and regional levels.

**Objectives**: We aimed to explore annual changes in cause-specific mortality at the country level, assess which causes contributed the most to child mortality reduction in 2000–2015, and estimate how many child lives were saved.

**Methods**: We used the cause-specific child mortality estimates published by Liu and colleagues. We derived average annual changes in cause-specific child mortality rates and cause-specific contribution to overall child mortality in 2000–2015. We estimated the number of cause-specific child deaths averted during the MDG era, assuming that cause-specific child mortality remained the same as in 2000. We targeted the 75 Countdown countries where 95% of maternal and child deaths occurred during the MDG era.

**Results**: Wide disparities existed across causes within countries, both in neonatal and post-neonatal mortality reduction, except for a few countries such as China, Rwanda, and Cambodia. In 20 of the 45 sub-Saharan African countries, malaria was the main contributor to post-neonatal mortality reduction, and pneumonia was the main contributor in only six countries. A single disease often contributed to a substantial proportion of the child mortality reduction, particularly in west and central African countries. Diarrhea-specific post-neonatal child mortality reduction accounted for 7.1 million averted child deaths (24.5%), while pneumonia accounted for another 6.7 million averted child deaths (23%).

**Conclusions**: This study demonstrates country-specific characteristics with regards to cause-wise child mortality that could not be identified by global or regional analyses. These findings provide the global community with evidence for formulating national policies and strategies to achieve the Sustainable Development Goals in child mortality reduction.

## Background

During the Millennium Development Goal (MDG) era, impressive reductions in the under-5 mortality rate (U5MR) have been observed, although the MDG 4 target was not met. The distribution of causes of child mortality has been updated, including the progress toward the MDG 4 target, at global, regional, and national levels [1–10].

The Sustainable Development Goals (SDGs) target an U5MR of no more than 25 per 1000 live births in every country of the world in 2030 [11]. While seeking to build upon the foundation laid by the MDGs to complete the unfinished business, we require evidence regarding what went well and what did not, which will guide and motivate future actions and constitute a set of priorities for child mortality reduction during the SDG era. In particular, to develop future strategy to achieve the SDG targets for U5MR, we should not miss the opportunity to assess cause-specific performance during the MDG era. So far, the main focus of the Global Burden of Disease Study [6–8,12] (GBD) has been to examine disease burden in terms of magnitude, distribution, and comparison by region or by year, while the cause-specific mortality fraction among overall child deaths and annual changes were mainly investigated for the overall U5MR. Recently global communities have become increasingly interested in investigating cause-specific progress (i.e. the annual change in mortality reduction) [1,4]; however, such analyses have been restricted to the global or regional level, and have not been conducted at the country level. Furthermore, some studies [1,2] have investigated cause-specific annual changes by U5MR strata (cutoffs: very high mortality stratum, 100 per 1000 live births; high, 75; medium-high, 50; medium, 25; low, 10; very low, less than 10). Prior studies [1,4,7] examining the leading causes and their overall progress at the global level, in specific regions, or across mortality strata might not have revealed features of nation-specific progress if national progress was not homogenous within the group. Furthermore, the results at the global level, in specific regions, or across mortality strata might have been disproportionally affected by a small number of countries with the greatest burden of child deaths, such as India, Ethiopia, Pakistan, and Nigeria. There has been substantial variation in investments in U5MR reduction in 2000–2015 by disease [1]. Understanding the impact of these investments on cause-specific mortality is required at the country level, considering the implications for national priorities. If the SDG targets for child survival are to be achieved, the corresponding policy priorities and future interventions should largely be developed at the country level. We thus aimed to investigate national cause-specific annual changes to help derive insights with implications for nation-specific policy.

A recent GBD study [7] investigated the attribution of changes in under-5 mortality to changes in the major causes of under-5 death; however, the target period in the study was 1990–2015, not 2000–2015. Trends in child mortality substantially changed in the early 2000s, when the MDG campaign and a number of global health initiatives were launched [8]. For example, HIV/AIDS- and malaria-specific under-5 deaths peaked in 2003 and 2005, respectively, and then decreased [8]. Since the relative distribution of child mortality by cause substantially differed between 1990 and 2000, we believe that the year 2000 is a more appropriate starting point for calculating the contribution of each cause to overall child mortality reduction. Moreover, we believe that 2000 is a particularly suitable starting point to derive implications for formulating future policy in the transition period from the MDG era to the SDG era, since an analysis starting in 2000 would allow us to better understand the performance of global efforts in reducing child mortality, particularly those made during the MDG campaign period. Using 1990 as the baseline year, as in the previous study [7], could lead to underestimation or overestimation of the contributions of certain causes, which eventually would generate misleading interpretations of investment in the past or misleading guidance for future policy. In addition, the previous study [7] calculated absolute values of reductions in cause-specific mortality. Since the leading causes of child mortality in the baseline year might have considerably affected the absolute values, this method makes it difficult to compare cause-specific performance between countries. Accordingly, we chose to assess the relative contributions of cause-specific mortality reductions to overall child mortality reduction in 2000–2015 at the country level, because we believe that doing so would yield more meaningful implications for policy formulation. In addition, evidence regarding the number of child lives saved by cause-specific performance is not only crucial for policy discussions and priority-setting, but is also important in terms of accountability, because it could have cost implications based on a comparison with the amount that the global ^14^community has invested in addressing specific diseases during the MDG era. We aimed to estimate the number of cause-specific child deaths averted in 2000–2015.

## Methods

### Data source

Annual assessments of trends in child mortality have been produced by the GBD and the UN Inter-agency Group for Child Mortality Estimation (IGME) using distinct methods [2,4,9,13–16], both of which are frequently used for understanding child mortality estimates and are highly correlated (0.983) [7]. In this study, we used the data published by Liu and colleagues [1] because the average annual changes of cause-specific child mortality have been examined using their dataset and method, although previous analyses were carried out at the global and regional levels. The number of cause-specific child deaths, both for neonates and children aged 1–59 months, were updated by Liu and colleagues in 2016 [1]. The number of age-specific deaths was multiplied by case-specific mortality fractions to estimate cause-specific child deaths. They used age-specific child mortality estimates produced by the UN IGME [17], and estimates of live births made by the UN Population Division [18]. To generate cause-specific child mortality fractions, only vital registration (VR) data were used for some countries (VR countries: 67 countries for neonates, 69 countries for 1- to 59-month-olds). A VR-based multi-cause model (VRMCM) was used for countries with inadequate VR and a low U5MR (less than 35 child deaths per 1000 live births in 2000–2015; 47 countries for neonates and 44 countries for 1- to 59-month-olds). For countries with inadequate VR and a high U5MR (35 child deaths or more per 1000 live births in 2000–2015; 80 countries for neonates and 81 countries for 1–59-month-olds), a verbal autopsy (VA)-based multi-cause model (VAMCM) was applied to estimate cause-specific child deaths. When estimating child deaths, they updated the database to include new VR data up to July 2015 and new VA data up to February 2015. The details of the estimation approaches developed by Liu and colleagues have been described elsewhere [1,4,9,19].

### Target countries

We targeted the 75 Countdown countries where 95% of maternal and child deaths occurred during the MDG era. The global community has recently expanded its attention to 81 countries for the SDG era [20,21], but we maintained the current list of 75 target countries because they are the group that received attention in 2000–2015, and most of the reports assessing MDG performance have focused on these 75 countries.

### Cause-specific annual changes in neonatal mortality and post-neonatal mortality

The average annual change in cause-specific mortality rates was derived using the following formula:

$$r=\left(ln\left(CSMR_{T2}/CSMR_{T1}\right)/\left(T2-T1\right)\right)*100$$

Where r is the average annual change of reduction, ln denotes the natural log function, CSMR is cause-specific mortality, and T1 and T2 denote the time points T1 and T2, respectively. A detailed explanation of the equation has been presented elsewhere [10,22]. We restricted the data analysis to 2000–2015 because the progress in child survival has been in part credited to the MDG campaign, which has led to the scaling-up of many life-saving interventions and increases in official development assistance [19,23–27].

### Categorization of progress

Based on the annual rate of reduction, we categorized progress into ‘success’ (average annual change of 4.4% or above; colored in green), ‘insufficient progress’ (1% to 4.3%, yellow), ‘no progress’ (0% to 1%, grey), and ‘negative (increase in mortality rate, red). We referred to the categories developed by the Countdown group [28].

We adjusted the success category to have a threshold of 4.4%, instead of the 4% threshold used by the Countdown group, because this was the minimum average change needed to meet the MDG 4 target. That is, child mortality should be reduced by 4.4% every year compared to the previous year if a country is to meet the goal of a two-thirds reduction of child mortality by 2015. The 1% criterion for ‘no progress’ was taken from the cut-off point used by the Countdown group, and 4.3% was purposefully adjusted from the 3.9% used in the Countdown group reports [23,29,30], taking into account the 4.4% cut-off point for success in this study. We also added the new category of ‘negative,’ which was used to indicate an increase in mortality.

### Number of cause-specific child deaths averted

We estimated the number of cause-specific child deaths averted during the MDG era referring to the methods to calculate all-cause child deaths averted in the previous study [2,31]. You and colleagues [2] estimated the number of all-cause child deaths averted in 2000–2015. Murray and Chambers [31] estimated the number of child lives saved by channel-specific spending on child health in 2000–2014. To estimate the number of averted child deaths in 2000–2015, two main methods can be used: one is to compare the values predicted on the basis of historical trends with those estimated on a yearly basis using the published data [1], and another is to compare the values assumed on the basis of child mortality remaining the same as in the year 2000 with those estimated on a yearly basis using published data [1]. We used the second method, assuming that cause-specific child mortality remained the same as in the year 2000, because there were no cause-specific child mortality data before 2000.

### Contribution of specific causes to overall child mortality

We explored to what extent each cause-specific mortality reduction contributed to overall child mortality reduction in 2000–2015, by dividing reduced all-cause child mortality by reduced cause-specific child mortality. We ranked the causes within each country by the percentage of contribution and explored whether there were any specific patterns by region.

## Results

In 2000–2015, the majority of Countdown countries (68.0%) showed a successful annual average reduction in post-neonatal mortality (in children aged 1–59 months), whereas only 6.7% had annual average changes of 4.4% or above in neonatal mortality (Table 1). Notably, for pneumonia, unlike the substantial proportion of countries with successful performance among post-neonatal children, only 25.3% (19 of 75) of the Countdown countries succeeded in meeting the anticipated annual changes in neonatal mortality. More than 80% of the countries showed successful annual reductions in post-neonatal mortality for meningitis (81.3%), diarrhea (82.7%), measles (89.3%), and malaria (85.3%). Although slow progress or unmet MDG targets could be mainly attributable to neonatal mortality, the majority of the Countdown countries made substantial progress in cause-specific neonatal mortality reductions for tetanus (94.7%) and diarrhea (78.7%).

**Table 1.** Overall results of performance in cause-specific child mortality reduction among 75 countries (neonates & 1–59-month-olds).

			Disease											
progress^a^	U5D^b^	POS^c^	PNE^d^	PRE^e^	INT^f^	MEN^g^	OTH^h^	CON^i^	DIA^j^	MEA^k^	INJ^l^	MAL^m^	AIDS^n^	PER^o^
Success (>4.4%)	49.3%(37/75)	68.0%(51/75)	64.0%(48/75)	17.3%(13/75)	16.0%(12/75)	81.3%(61/75)	25.3%(19/75)	12.0%(9/75)	82.7%(62/75)	89.3%(67/75)	9.3%(7/75)	85.3%(64/75)	56.0%(42/75)	1.3%(1/75)
Insufficient (1.0%-4.4%)	50.7%(38/75)	32.0%(24/75)	30.7%(23/75)	46.7%(35/75)	46.7%(35/75)	17.3%(13/75)	41.3%(31/75)	41.3%(31/75)	17.3%(13/75)	5.3%(4/75)	44.0%(33/75)	12.0%(9/75)	16.0%(12/75)	68.0%(51/75)
No progress (0%-1.0%)	0%	0%	4.0%(3/75)	17.3%(13/75)	14.7%(11/75)	1.3%(1/75)	12.0%(9/75)	20.0%(15/75)	0%	1.3%(1/75)	13.3%(10/75)	1.3%(1/75)	5.3%(4/75)	26.6%(20/75)
Increase (negative)	0%	0%	1.3%(1/75)	18.7%(14/75)	22.7%(17/75)	0%	21.3%(16/75)	26.7%(20/75)	0%	4.0%(3/75)	33.3%(25/75)	1.3%(1/75)	22.7%(17/75)	4.0%(3/75)

		Disease							
	NEO^p^	PNE	PRE	INT	SEP^q^	TET^r^	OTH	CON	DIA
Success (>4.4%)	6.7%(5/75)	25.3%(19/75)	16.0%(12/75)	20.0%(15/75)	5.3%(4/75)	94.7%(71/75)	13.3%(10/75)	1.3%(1/75)	78.7%(59/75)
Insufficient (1.0–4.4%)	86.7%(65/75)	4.0%(3/75)	76.0%(57/75)	76.0%(57/75)	62.7%(47/75)	4%(3/75)	69.3%(52/75)	14.7%(11/75)	18.7%(14/75)
No progress (0–1.0%)	5.3%(4/75)	69.3%(52/75)	5.3%(4/75)	1.3%(1/75)	17.3%(13/75)	0%	14.7%(11/75)	21.3%(16/75)	1.3%(1/75)
Increase (negative)	1.3%(1/75)	1.3%(1/75)	2.7%(2/75)	2.7%(2/75)	14.7%(11/75)	1.3%(1/75)	2.75(2/75)	62.7%(47/75)	1.3%(1/75)

^a^green: success (average annual change of 4.4% or above); yellow: insufficient progress (1% to 4.3%); grey: no progress (0% to 1%); red: negative (increase in mortality rate); N/A: <1death per 1000 live births in 2000; ^b^under-5 child; ^c^post neonatal; ^d^pneumonia; ^e^preterm birth complication; ^f^intrapartum-related events; ^g^meningitis; ^h^other diseases; ^i^congenital anomaly; ^j^diarrhea; ^k^measles: ^l^injuries; ^m^malaria; ^n^AIDS; ^o^pertusis; ^p^neonatal; ^q^sepsis; ^r^tetanus.

**Figure 1.**
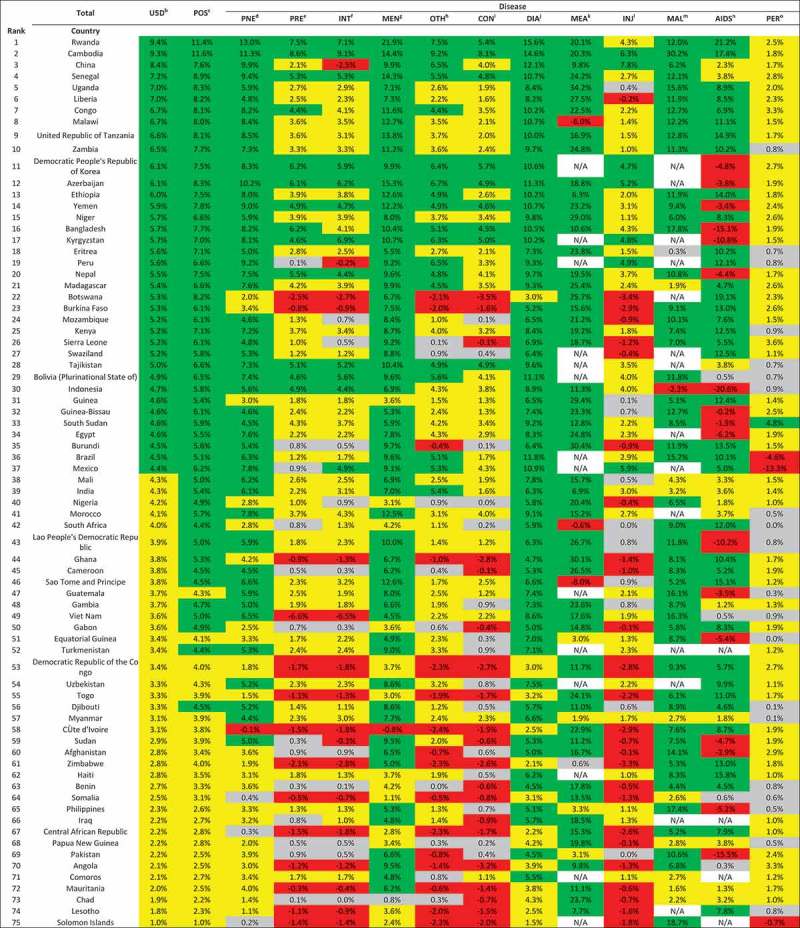
Average annual change^a^ of cause-specific child mortality at country level for 75 countries (1–59-month-olds).

**Figure 2.**
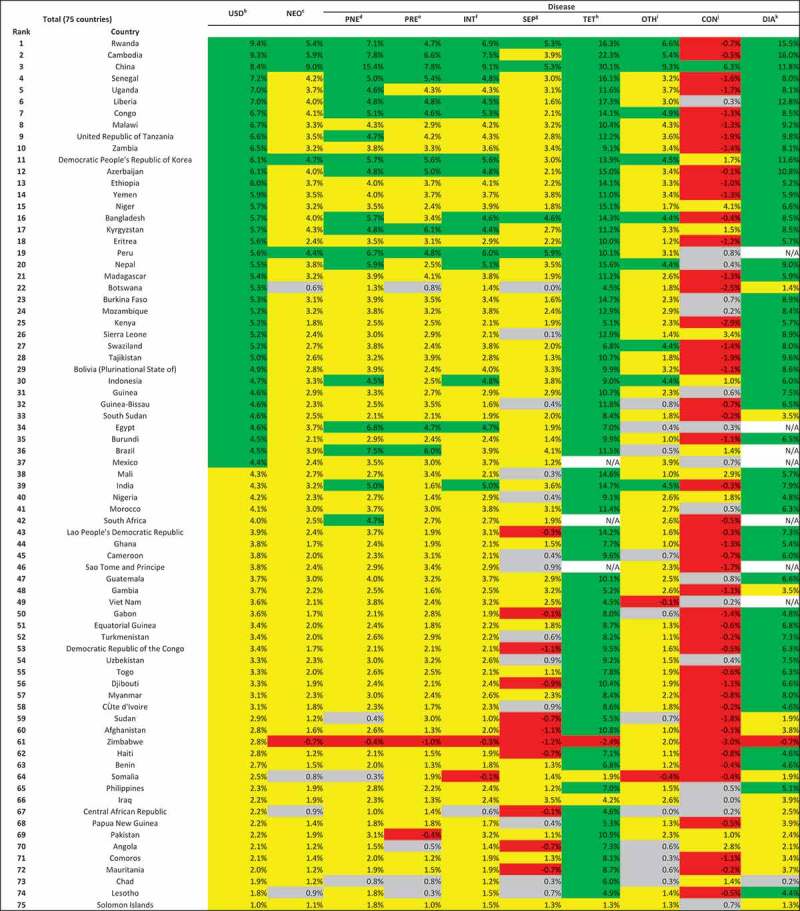
Average annual change^a^ of cause-specific neonatal mortality at country level for 75 countries.

Figures 1 and 2 reveal that wide disparities existed across causes within countries, both in neonatal and post-neonatal mortality reduction. China is the only country that successfully reduced neonatal mortality from every cause. Except for a few countries such as China, Rwanda, and Cambodia, the annual changes in neonatal mortality varied to a large degree. In Zimbabwe, the cause-specific neonatal mortality rate increased during this period for 7 causes.

**Figure 3.**
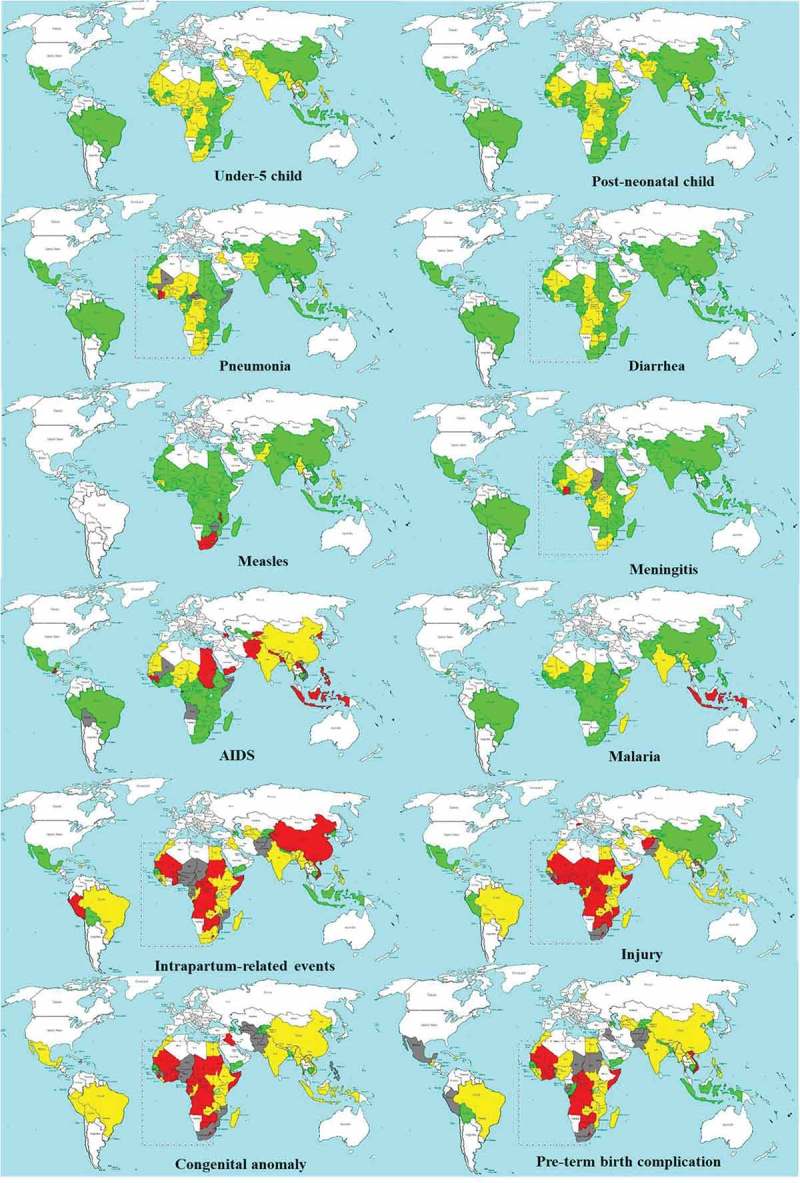
Overall and cause-specific child mortality progress of 75 countries in 2000–2015 (1–59-month-olds, green: success, average annual change of 4.4% or above; yellow: insufficient progress, 1% to 4.3%; grey: no progress, 0% to 1%; red: negative, increase in mortality rate).

Particular attention should be given to certain countries with an increase of cause-specific mortality even for these infectious diseases (Figure 3). For example, Malawi saw increased measles-specific child mortality (−6%), as did Cote d’Ivoire for meningitis (−0.8%). There is no country that successfully reduced all cause-specific child mortality rates among post-neonatal children. Cambodia was the only country among this group to show successful annual reductions in 11 of the 12 causes. Ranks of annual change by region are shown in Appendix 1 and 2.

**Figure 4.**
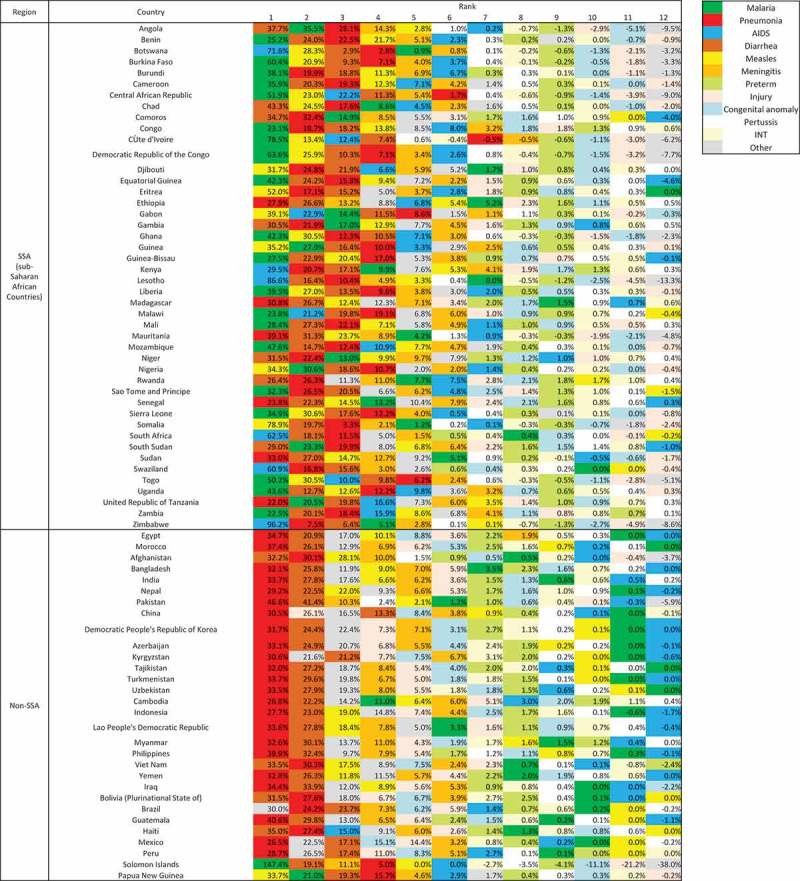
Cause-specific contribution to overall child mortality at country level for 75 countries (1–59-month-olds).

**Figure 5.**
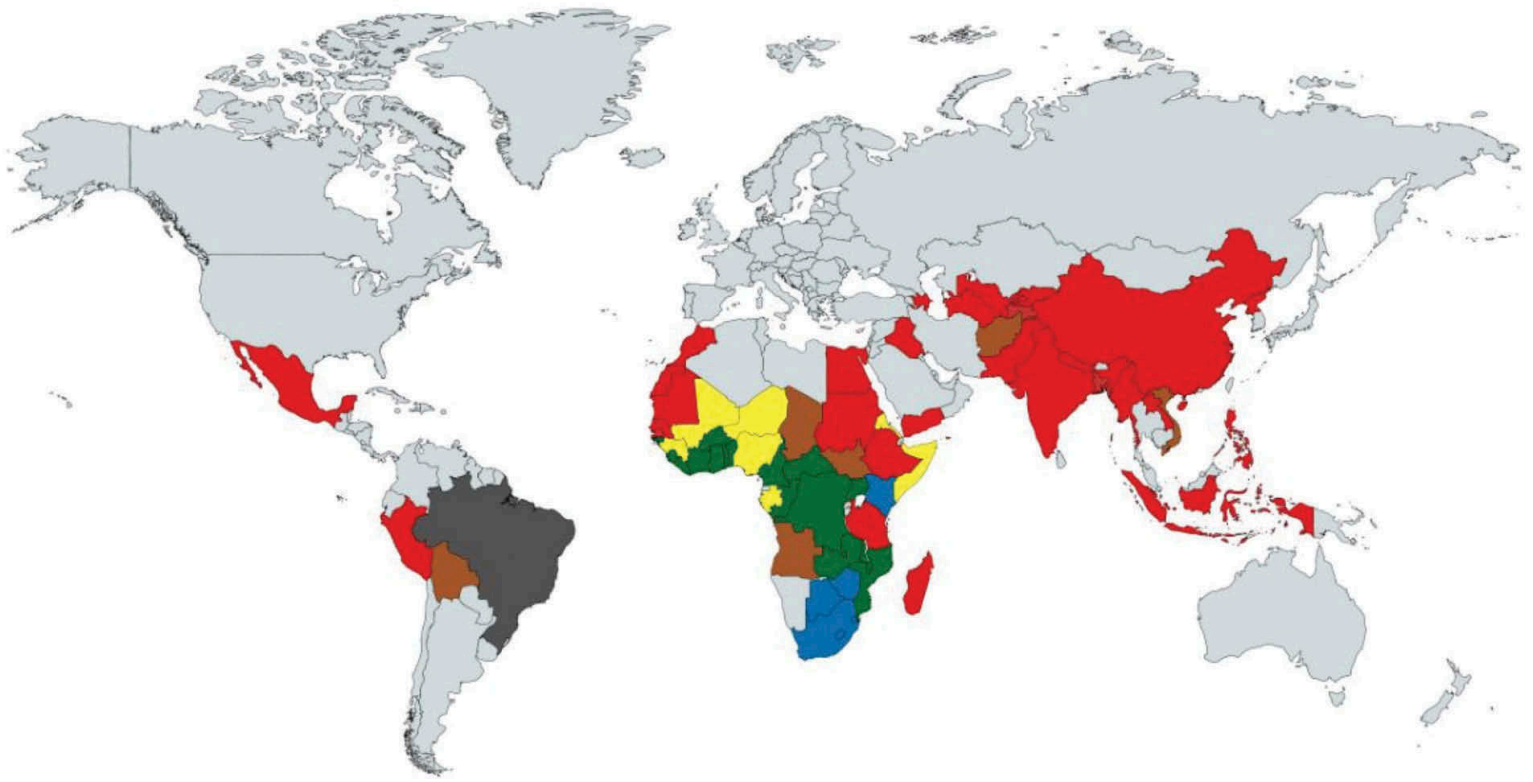
Key contributor of under-5 child mortality reduction of 75 countries in 2000–2015 (green: malaria; blue: AIDS; red: pneumonia; yellow: measles; brown: diarrhea; grey: other diseases).

Table 2, Figure 4, and Appendix 3 show cause-specific contributions to overall post-neonatal and neonatal mortality reductions. A clear distinction can be seen between sub-Saharan African countries and other regions in terms of the main contributors to post-neonatal child mortality reduction. In 20 of the 45 sub-Saharan African countries, malaria was the main contributor to post-neonatal mortality reduction (Figure 5). AIDS contributed the most to child mortality reduction in six countries that had an extremely high prevalence in 2000, ranging from a contribution of 86.6% of the all-cause child mortality reduction in Lesotho to 29.5% in Kenya. In other regions, pneumonia was the main contributor to child mortality reduction, ranging from a contribution of 46.6% of the all-cause child mortality reduction in Pakistan to 26.8% in Cambodia. Pneumonia was the main contributor in only 6 countries and the second contributor in 12 countries among the sub-Saharan African countries, although it accounted for a large proportion of child mortality in 2000.

**Table 2.** Main contributors to overall child mortality at country level for 75 countries (1–59-month-olds).

		Total						Sub-Saharan African countries (SSA)						Non-SSA					
Disease		Rank 1		Rank 2		Rank 3		Rank 1		Rank 2		Rank 3		Rank 1		Rank 2		Rank 3	
	Malaria	21	28.0%	6	8.0%	4	5.3%	20	43.5%	5	11.1%	4	8.9%	1	3.3%	1	3.3%	0	0.0%
	Pneumonia	29	38.7%	17	22.7%	13	17.3%	6	13.0%	12	26.7%	13	28.9%	23	76.7%	5	16.7%	0	0.0%
	AIDS	6	8.0%	2	2.7%	4	5.3%	6	13.0%	2	4.4%	3	6.7%	0	0.0%	0	0.0%	1	3.3%
	Diarrhea	11	14.7%	36	48.0%	22	29.3%	6	13.0%	16	35.6%	17	37.8%	5	16.7%	20	66.7%	5	16.7%
	Measles	7	9.3%	10	13.3%	12	16.0%	7	15.2%	10	22.2%	6	13.3%	0	0.0%	0	0.0%	6	20.0%
	Meningitis	0	0.0%	0	0.0%	3	4.0%	0	0.0%	0	0.0%	1	2.2%		0.0%	0	0.0%	2	6.7%
	Preterm	0	0.0%	0	0.0%	0	0.0%	0	0.0%	0	0.0%	0	0.0%		0.0%	0	0.0%	0	0.0%
	Injury	0	0.0%	1	1.3%	0	0.0%	0	0.0%	0	0.0%	0	0.0%		0.0%	1	3.3%	0	0.0%
	Congenital anomaly		0.0%		0.0%		0.0%		0.0%		0.0%		0.0%		0.0%	0	0.0%		0.0%
	Pertussis		0.0%		0.0%		0.0%		0.0%		0.0%		0.0%		0.0%	0	0.0%		0.0%
	Intrapartum related events		0.0%		0.0%		0.0%		0.0%		0.0%		0.0%		0.0%	0	0.0%		0.0%
	Other	1	1.3%	3	4.0%	17	22.7%	0	0.0%	0	0.0%	1	2.2%	1	3.3%	3	10.0%	16	53.3%

**Figure 6.**
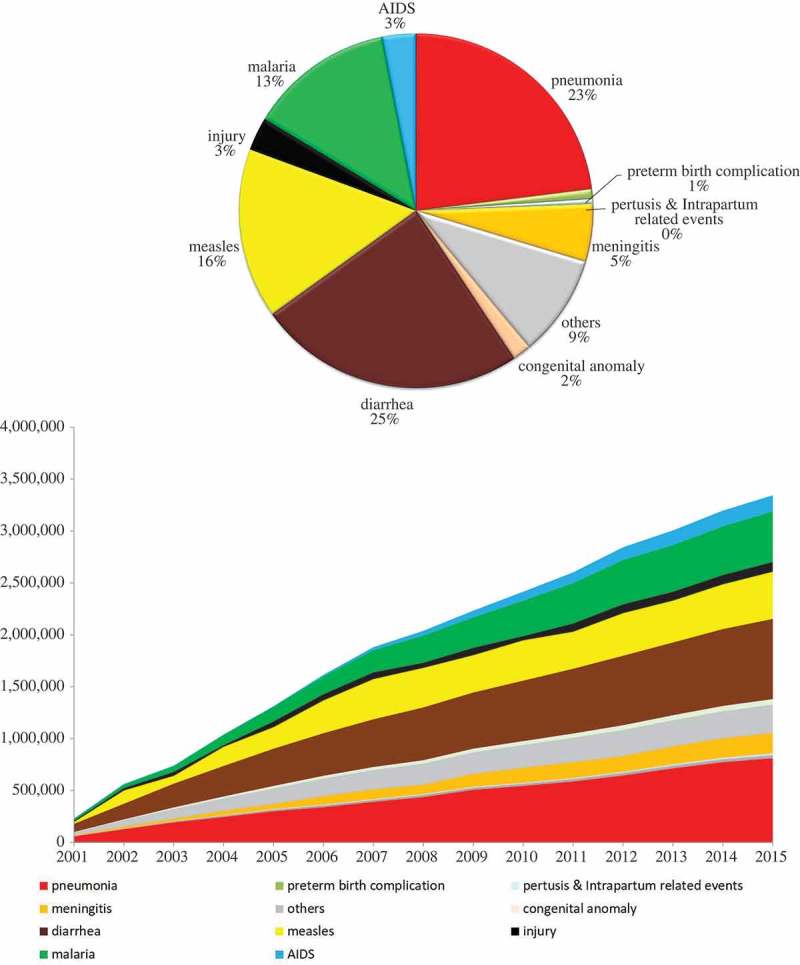
Number of child lives saved during the MDG era by disease among 75 countries (1–59-month-olds, above: accumulated number of lives saved; below: annual number of lives saved).

**Figure 7.**
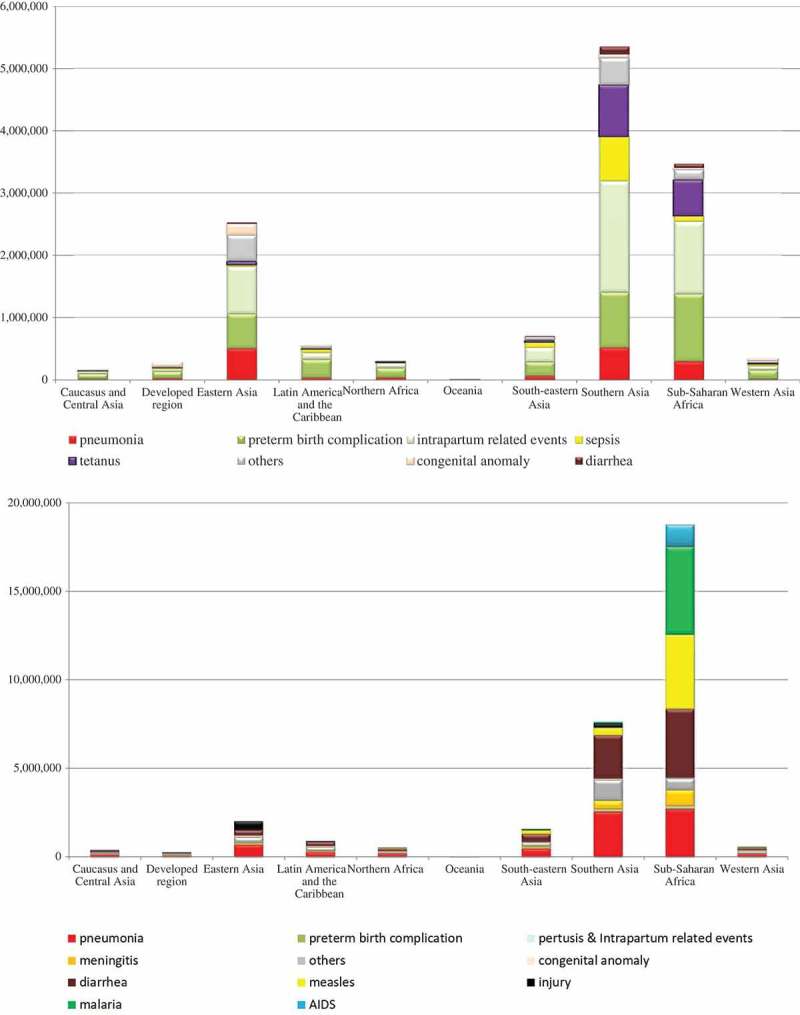
Number of cause-specific child deaths averted during the MDG era by region (above: neonates; below: 1–59-month-olds).

During the MDG era, 42.7 million child deaths were averted; the majority of the lives saved took place in sub-Saharan Africa for post-neonatal children and in southern Asia for neonates (Figure 6–7, Appendix 4 and 5). Of these averted child deaths, 13.7 million were in neonates and 29 million were in post-neonatal children. Diarrhea-specific post-neonatal child mortality reduction accounted for 7.1 million averted child deaths (24.5%), while pneumonia accounted for another 6.7 million averted child deaths (23%) (Figure 6, Appendix 4). Tetanus-specific neonatal mortality reduction averted 1.5 million neonatal deaths. Considering the disease burden of pneumonia and diarrhea among children, interventions related to diarrhea performed much more successfully than those for pneumonia.

## Discussion

During the MDG era, a tremendous number of children’s lives were saved, and this has been partially credited to the global efforts following the MDGs, the ensuing increase in official development assistance [19], and the consequential scaling up of many life-saving interventions [21]. This study suggests that the progress has been uneven across diseases within countries, even in countries known as the best performers, such as Botswana or Burkina Faso, which have made successful progress in overall child mortality reduction. In the past, pneumonia, malaria, and diarrhea were the leading causes in the very-high-mortality stratum [7], to which Angola, Central African Republic, Chad, Mali, Nigeria, Sierra Leone, and Somalia belong; however, according to our results, cause-specific progress for these main infectious diseases substantially varied by country. For example, Liu and colleagues [1] pointed out that tremendous progress has been made in diarrhea, pneumonia, measles, and neonatal tetanus. They underscored that five of the 12 post-neonatal causes achieved a sufficient annual reduction (≥4.4%); however, the insufficient progress in pneumonia- and diarrhea-specific child mortality, particularly among west and central African countries (Zimbabwe, Central African Republic, Lesotho, Democratic Republic of Congo, South Africa, Togo, Chad) have not previously been emphasized. If pneumonia and/or diarrhea had declined at the rate achieved for other infectious diseases, such as malaria, measles, meningitis, and AIDS, many of these countries might have attained the MDG 4 target. Similarly, the leading causes in the high, medium-high, and medium mortality strata, to which 17, 20, and 36 countries belong, respectively, were found to be similar (preterm birth complications, pneumonia, and intrapartum-related events), but the annual change of cause-specific child mortality differed considerably within the same strata. These findings have never been reported in prior studies, since previous analyses focused on global, regional, and national causes of under-5 mortality [6–8,32–34]. Additionally, it is noteworthy that malaria-specific child mortality reduction was a key contributor to overall child mortality reduction in sub-Saharan Africa, while pneumonia was a key contributor in other regions among the Countdown countries during the MDG campaign. The efforts of the global community to combat malaria [35] can be given credit for this significant contribution. In contrast to the study of Wang and colleagues [13], which highlighted a rapid decrease in child mortality subsequent to the scale-up of ART and prevention of mother-to-child transmission, AIDS was not the largest contributor to child mortality reduction in the sub-Saharan African region, either at the country level (with some exceptions) or at the regional level.

It is already well known that neonatal mortality has declined more slowly than post-neonatal child mortality [28]. However, looking into the progress by cause, some areas of cause-specific neonatal mortality reduction, such as diarrhea and tetanus, have seen enormous success in many countries. The more rapid reduction of child mortality in post-neonatal children than in neonates was mainly due to reductions in the burden of infectious disease. Among post-neonatal children, many countries saw insufficient progress in preterm birth complications, congenital anomalies, injuries, and intrapartum-related events

Caution is needed when interpreting the relatively low percentage of countries that were successful with AIDS and pertussis, because these results may have primarily been due to very low child mortality rate of many countries for these diseases in 2000, the baseline year of the comparisons.

Although 27 countries achieved success in overall child mortality reduction in terms of the annual average changes set by the MDGs, none of the countries saw success in all 12 causes in post-neonatal children. Some exceptional countries such as China, Rwanda, and Cambodia achieved an impressive and balanced decline across causes. West and central Saharan Africa had the largest disparities both in terms of progress and contributions. Attention should be paid to the finding that in this group of countries, a single disease often contributed to a substantial proportion of the child mortality reduction. For example, in Lesotho, South Africa, and Zimbabwe, AIDS-specific child mortality reduction alone contributed to 60–95% of the all-cause child mortality reduction, and malaria contributed to 50–80% of the reduction in Cute d’Ivoire, Democratic Republic of the Congo, Burkina Faso, Central African Republic, and Togo. Another example is Botswana, which is frequently referred as a successful country [12] in terms of child mortality reduction during the MDG campaign, as it showed 5.3% annual changes; however, 71.6% of the child mortality reduction in Botswana can be attributed to AIDS-specific child mortality reduction alone.

It is worrisome that successful performance in a very specific disease could mask unsuccessful progress with other diseases, and that success in terms of the annual average reduction in all-cause child mortality could misrepresent poor performance in certain areas of cause-specific mortality reduction within the same country if we do not pay attention to cause-specific progress and contributing factors at the country level [15].

Additionally, the specific characteristics of west and central African countries should be noted. Despite considerable reductions in overall infectious disease mortality across 75 countries, progress in pneumonia- and diarrhea-specific child mortality was not sufficient in west and central African countries, in contrast with east Africa. Thirty percent (1.8 million) of worldwide child deaths took place in west and central Africa in 2015, and six of the seven countries with a U5MR over 100 per 1000 live births are in this area (Angola, Chad, Central African Republic, Sierra Leone, Mali, and Nigeria) [2]. You and colleagues predicted the number of global under-five deaths would be 6.6 million in 2030, higher than that in 2015, under the scenario of no change from the 2015 mortality rate, and they reported that the increase would be largely attributable to the growing under-five population in west and central Africa [2]. Prior studies [1,7] have stressed that low-income countries should accelerate progress, particularly for neonates; however, insufficient progress in pneumonia and diarrhea in west and central Africa, and some outliers in cause-specific child mortality reduction (e.g. increases in pneumonia and meningitis in Cute d’Ivoire, measles in South Africa and Malawi) have not been duly highlighted. The relatively even progress across causes of child mortality seen in Rwanda, Cambodia, China, and many east African countries should be further examined in future research.

Examining the specific reasons for the uneven or even progress seen in specific countries is beyond the scope of this study; however, we present some possible factors that require further investigation. The first factor is the overall strength of the health system [36,37]. While measles and tetanus-specific deaths can be prevented by relatively simple interventions, such as immunization [38,39], more comprehensive interventions are needed to prevent deaths from pneumonia and diarrhea, involving more a comprehensive approach, including behavior change, inter-sectoral collaboration, and environmental improvements [39–41], all of which require a strengthened health system [42,43]. We suppose that a substantial share of specific causes in terms of contribution might have been attributed to some vertical programs generating considerable investment in specific diseases as part of global health initiatives around 2000 [36]. If successful results can be caused by a vertical program with nothing to do with overall strengthening of the health system, we cannot be sure whether the accelerated progress seen in 2000–2015 could be pronounced and sustained for other diseases within the same country during the SDG era. The second factor is inequality in health coverage [44–46]. It is noteworthy that the degree of inequality in coverage of interventions for child health was much smaller in Cambodia and Rwanda, both of which saw successful progress across causes, in contrast with the considerable inequality seen in Angola, Nigeria, and the Central African Republic [21]. According to the results of GBD, China, also one of the best performers, was reported to have equal progress across provinces and municipalities. A third factor is war or internal conflict [47–49]. Internal conflicts or war might have escalated all or some causes of child mortality in some countries (e.g. Cute d’Ivoire, Somalia, Sudan, Democratic Republic of Congo).

A clear child survival policy and program focus should be based on the performance in the last 15 years and the contributions of disease-specific reduction to all-cause child mortality, in order for interventions to have the potential to further improve overall child survival. In this regard, although the global community has tended to shift its attention toward neonatal mortality reduction [21,50–54], the focus should still be on the leading infectious causes of post-neonatal mortality in many countries, especially in west and central African countries, for child survival efforts. This is warranted because the substantial contribution of one or a few cause-specific reductions may mask unsuccessful performance in other areas of cause-specific child mortality reduction.

The main limitation of this study lies in its data source, since there is a large data gap for these countries. Our study was based on data published by Liu and colleagues [1], who clearly stated that inherent uncertainties and limited misclassifications exist in the classification of VAs in the current data, and the estimation of cause-specific mortality is especially hindered by data limitations because a minimal proportion of the global cases of mortality in under-5 children are medically certified. In low- or middle-income, child mortality is based on a comparably large amount of empirical data to what is used for adult or old-age mortality, although data quality issues still pose challenges in many countries. To create reliable and comparable child mortality estimates, modeling exercises have been conducted and continue to be refined. For most of the target countries in this study, cause-specific child deaths were estimated by VRMCM or VAMCM, with VA used for 37 countries for neonates and 42 countries for 1- to 59-month-olds, and VR for 66 countries for neonates and 68 countries for 1- to 59-month-olds. Although the accuracy of VA or VR remains to be improved, the increased number of data points (i.e. 2004 data point for VR for neonates and 1364 for 1-to 59-month-olds; 124 for VA for neonates and 218 for 1- to 59-month-olds in their updated report in 2016) ensure greater validity than previous results, and these estimates have been widely used by diverse organizations, including the World Health Organization.

Roughly 43 million child deaths have been avoided during the MDG era in 75 countries. The finding that a substantial number of child lives saved occurred in sub-Saharan Africa for post-neonatal children, and in South Asia for neonates, is plausible in light of the fact that the older group has seen twice the progress of neonates in the sub-Saharan Africa region in 2000–2015 [2]. The substantial portion of averted child deaths from neonate tetanus and measles, particularly in sub-Saharan Africa, should be highlighted. The lower proportion of averted post-neonatal child deaths from pneumonia compared with diarrhea warrants further research, since pneumonia is responsible for a larger proportion of child deaths; this discrepancy may suggest there is more room for improvement in this leading cause of child death. The assumption that the child mortality remained the same throughout the study period, which we used to derive the number of child lives saved, might have led to either an overestimation of overall under-5 child lives saved since the trend was already decreasing or an underestimation of averted child deaths from some other causes, such as HIV/AIDS and malaria, which showed increasing trends around the year 2000. However, all in all, the share of child lives saved is in line with prior studies [2,31] that reported that 48 million lives were saved in 2000–2015 and 37 million in 2000–2013 among 195 countries; in that study, You and colleagues [2] suggested that 18 million of the child lives saved were attributed to the accelerated progress of child mortality reduction since 2000. For the upcoming period, we could estimate the number of cause-specific averted child deaths by applying various scenarios, such as no change from the 2016 mortality rate, current trends, and the trends reported by the best regional performer based on cause-specific progress in 2000–2015.

The results of our cause-specific analysis at the country level will guide further efforts at the global and country levels, and this study warrants future research that will provide evidence regarding the avoidability of cause-specific deaths for causes that did not show successful progress and still have substantial room for future contributions.

As a method of calculating average annual changes, we considered summing up every year’s change in 2001–2015 and then dividing the result by 15, but we eventually used the values derived from the formula provided presented in methods part for the following reasons. First, in some diseases (for example, measles), abrupt changes took place in some countries in certain years, affecting the mean values too much and potentially leading to a misrepresentation of the average change or general trend during the MDG era. Second, previous studies used the formula instead of calculating the average value by dividing the summed-up value by 15, and we therefore applied the same method to improve the comparability of our findings with those of previous studies. An uncertainty interval of 90% or 95% has been suggested for cause-specific child mortality or annual changes, and the uncertainty in model coefficients could be estimated by resampling the input data sets an enormous number of times. Since this study used already-generated mortality outcomes and we did not handle the input data, we were unable to carry out estimation procedures for uncertainty intervals. We suggest that future studies generating cause-specific child mortality estimates should derive uncertainty intervals for annual changes at the national level.

The global annual average rate of reduction (AARR) in mortality has steadily doubled after the Millennium Declaration, and many sub-Saharan African countries saw an average AARR of 4.4% or above in 2000–2015; however, further studies must be conducted to investigate whether these improvements were accompanied by strengthening of the overall health systems or were temporary trends subsequent to the rapid increase in vertical programs focused on those specific diseases.

## Conclusion

The SDGs aim to reduce U5MR to 25 child deaths or below per 1000 live births by 2030 in every country, and the cause-specific progress in 2000–2015 should be addressed as part of policy discussions, especially when formulating country-specific strategies. The leading infectious diseases such as pneumonia and diarrhea have long been suggested to be a policy priority going forward in very-high-mortality countries; however, only emphasizing these diseases is insufficient. Furthermore, a thorough investigation must be undertaken to examine the reasons for the slow reduction in these two main infectious diseases in west and central African countries, despite the existence of a range of highly cost-effective and low-cost interventions, such as vaccination for pneumonia, water and sanitation improvements, breastfeeding promotion and the like. We postulated that the overall strength of health systems, inequality, and war or conflict may be possible factors contributing to success or failure in reducing mortality due to these causes. Case studies on the remarkable progress shown for these two diseases in east African countries could generate clues about possible solutions suitable for neighboring regions. Many countries in west and central Africa have seen increases in pre-term birth complications, intra-partum related events, congenital anomalies, and injuries, all of which should be a focus of child survival efforts; however, investments in interventions against pneumonia and diarrhea must not be crowded out. Recommendations for policy formulations in prior studies have generally been derived from findings at global, regional, or U5MR-mortality stratum levels. These recommendations may have been insightful to some extent, insofar as some patterns do exist at those levels. However, analyses at the global level, in certain regions, or across certain mortality strata are susceptible to be disproportionately affected by a few countries with an enormous burden of child deaths. The results of this study support this argument by demonstrating the distinctive features that can be observed in a country-level analysis.

To identify potential challenges in achieving the SDG targets in child mortality reduction, lessons learnt from failure or successes should be shared. The progress in overall child mortality cannot itself indicate the gaps that remain to be filled in terms of investment, commitment, or prioritization; what went well or what went wrong in the MDG period; or what more we can do in the SDG era in each country. Such conclusions should be based on country-specific findings. We believe that our key findings with regard to cause-specific progress in each country provide insight into the formulation of country-specific strategies.

## Figures and Tables

**Figure:**
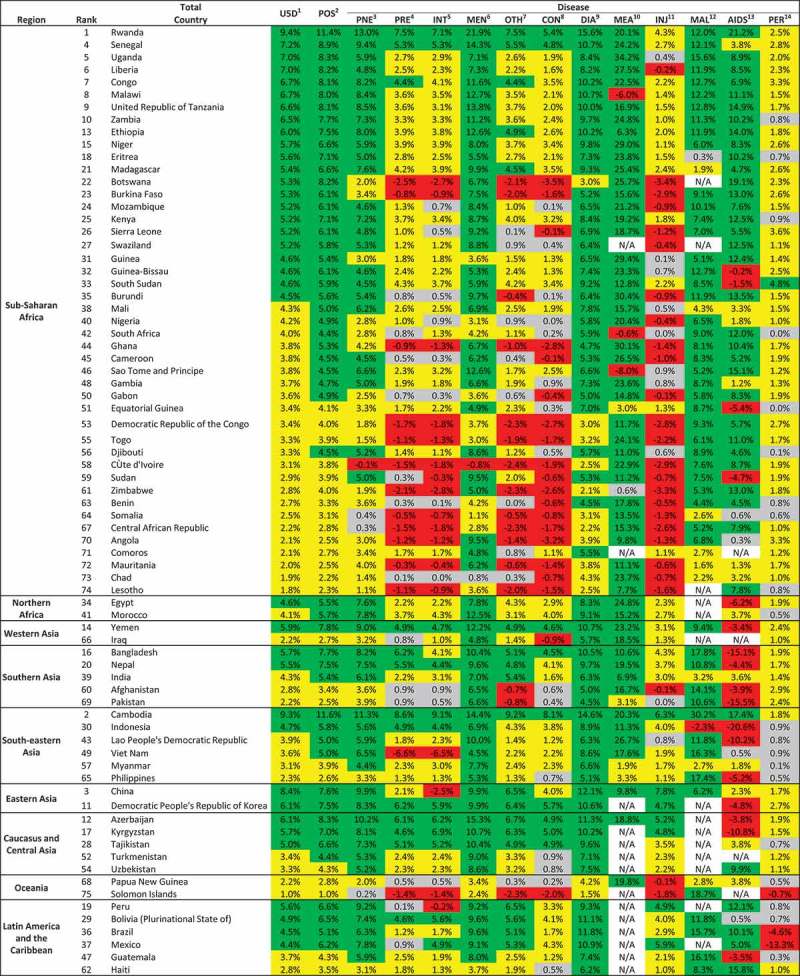
^a^Green: success (average annual change of 4.4% or above); yellow: insufficient progress (1% to 4.3%); grey: no progress (0% to 1%); red: negative (increase in mortality rate); N/A: <1death per 1000 live births in 2000; ^b^under-5 child ; ^c^post neonatal; ^d^pneumonia; ^e^preterm birth complication; ^f^intrapartum-related events; ^g^meningitis; ^h^other diseases; ^i^congenital anomaly; ^j^diarrhea; ^k^measles: ^l^injuries; ^m^malaria; ^n^AIDS; ^o^pertusis.

**Figure:**
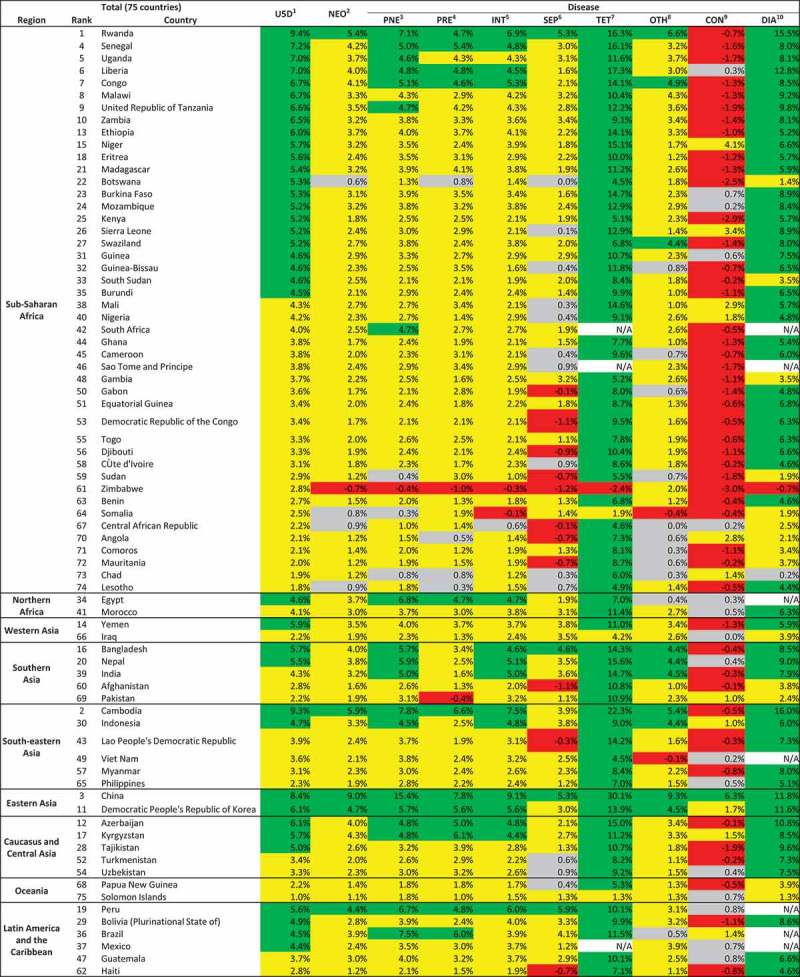
^a^green: success (average annual change of 4.4% or above); yellow: insufficient progress (1% to 4.3%); grey: no progress (0% to 1%); red: negative (increase in mortality rate); N/A: <1death per 1000 live births in 2000; ^b^under-5 child ; ^c^post neonatal; ^d^pneumonia; ^e^preterm birth complication; ^f^intrapartum-related events; ^g^meningitis; ^h^other diseases; ^i^congenital anomaly; ^j^diarrhea; ^k^measles: ^l^injuries; ^m^malaria; ^n^AIDS; ^o^pertusis.

## Paper context

Previously, performance in child mortality reduction has been assessed all-cause child mortality. Cause-specific changes in child mortality have been analyzed and discussed mainly at the global and regional levels. We assessed performance in cause-specific child mortality at the country level and analyzed which causes contributed the most to child mortality reduction in 2000–2015. In addition, we estimated the number of cause-specific child deaths averted during the MDG era, referring to the methods to calculate all-cause child deaths averted in the previous study. Governments have pledged US$ 25 billion for improving maternal, adolescent, and child health from 2016 to 2020, and this investment should be guided by lessons obtained from reflecting on past performance. This study provides meaningful insights into the formulation of country-specific strategies.

## Appendix 4. Number of child deaths averted during the MDG era by disease among 75 countries (1–59-month-olds)

**Table:**

Year	U5D^a^	POS^b^	PNE^c^	PRE^d^	INT^e^	MEN^f^	OTH^g^	CON^h^	DIA^i^	MEA^j^	INJ^k^	MAL^l^	AIDS	PER^m^
2001	345,935	224,819	59,033	2,612	783	6,166	27,924	4,169	77,230	18,308	12,110	20,967	−4,457	−25
2002	808,648	556,413	128,756	5,456	1,736	18,798	58,537	8,683	150,169	128,317	24,534	37,354	−5,844	−82
2003	1,119,949	734,789	193,500	9,585	3,151	22,889	91,963	16,248	227,563	75,665	39,031	59,165	−4,639	666
2004	1,557,796	1,038,677	245,659	11,940	3,942	41,547	119,179	20,098	292,932	185,489	16,508	100,551	−235	1,066
2005	1,952,605	1,311,977	301,407	15,028	4,944	47,870	148,551	25,630	357,647	207,830	56,427	138,430	6,711	1,502
2006	2,367,138	1,614,251	340,125	16,131	5,576	83,215	168,263	27,593	409,441	316,157	60,522	170,023	15,430	1,773
2007	2,732,253	1,880,153	391,594	17,405	6,404	94,567	185,657	29,849	458,579	387,643	65,934	212,945	27,861	1,715
2008	2,981,520	2,037,425	437,947	19,137	7,234	89,376	203,331	34,238	507,059	381,438	50,356	260,335	45,296	1,679
2009	3,263,721	2,231,731	507,736	19,284	7,157	124,589	207,240	36,071	539,860	359,757	72,371	292,751	63,026	1,888
2010	3,535,397	2,414,203	544,902	20,753	7,650	143,795	220,137	37,697	581,347	388,666	42,293	339,823	85,499	1,640
2011	3,818,422	2,600,308	588,026	22,322	8,446	153,488	234,508	42,054	622,699	356,693	80,889	387,159	103,021	1,003
2012	4,159,778	2,842,460	645,407	24,797	9,327	151,153	251,243	47,184	667,681	410,895	86,635	424,796	122,552	791
2013	4,424,731	3,004,602	714,256	25,653	9,120	172,323	251,459	49,859	699,399	403,675	86,640	448,424	140,340	3,451
2014	4,704,134	3,195,643	773,856	27,229	9,415	186,514	260,320	52,459	739,223	431,578	90,481	467,230	151,511	5,826
2015	4,930,074	3,343,154	811,480	28,993	10,251	195,149	273,096	54,816	770,853	455,266	95,645	485,165	154,670	7,768
total	42,702,102	29,030,604	6,683,683	266,323	95,135	1,531,438	2,701,408	486,648	7,101,682	4,507,376	880,376	3,845,119	900,740	30,662
			23.0%	0.9%	0.3%	5.3%	9.3%	1.7%	24.5%	15.5%	3.0%	13.2%	3.1%	0.1%

^a^under-5 child ; ^b^post neonatal; ^c^pneumonia; ^d^preterm birth complication; ^e^intrapartum-related events; ^f^meningitis; ^g^other diseases; ^h^congenital anomaly; ^i^diarrhea; ^j^measles: ^k^injuries; ^l^malaria; ^m^pertusis.

We estimated the number of cause-specific child deaths averted by comparing the number of cause-specific child deaths published and the estimates assuming that child mortality remain the same as in the year 2000 on a yearly basis.

## Appendix 5. Number of neonatal deaths averted during the MDG era by disease among 75 countries

**Table:**

Year	NEO^a^	PNE^b^	PRE^c^	INT^d^	SEP^e^	TET^f^	OTH^g^	CON^h^	DIA^i^
2001	121,116	14,009	28,992	34,720	8,378	19,576	9,770	3,405	2,266
2002	252,235	28,593	61,512	74,357	17,297	37,830	20,265	7,923	4,457
2003	385,160	42,354	93,307	116,347	27,227	54,210	31,939	13,242	6,533
2004	519,118	56,732	127,476	159,064	37,191	68,893	44,119	16,996	8,647
2005	640,627	71,128	154,803	198,516	47,579	80,968	56,571	20,412	10,651
2006	752,887	84,511	182,934	232,586	57,849	90,948	68,063	23,259	12,736
2007	852,100	96,427	206,406	264,415	64,946	99,117	78,593	27,591	14,605
2008	944,095	106,311	229,680	295,359	71,847	106,623	86,795	31,059	16,422
2009	1,031,989	115,344	251,075	325,167	80,839	113,126	94,538	33,745	18,154
2010	1,121,193	124,138	268,791	356,898	94,857	118,479	103,812	34,476	19,743
2011	1,218,114	132,645	289,155	389,822	113,350	123,211	113,143	35,455	21,334
2012	1,317,318	141,739	309,253	423,573	132,457	128,157	122,045	37,233	22,860
2013	1,420,129	150,805	333,260	455,675	151,098	133,329	131,551	39,975	24,436
2014	1,508,491	158,313	355,741	483,101	165,699	138,038	140,204	41,512	25,884
2015	1,586,920	165,023	376,602	506,623	179,471	141,947	147,242	42,905	27,107
Total	13,671,494	1,488,072	3,268,988	4,316,223	1,250,085	1,454,453	1,248,649	409,188	235,835
		10.9%	23.9%	31.6%	9.1%	10.6%	9.1%	3.0%	1.7%

^a^neonatal; ^b^pneumonia; ^c^preterm birth complication; ^d^intrapartum-related events; ^e^sepsis; ^f^tetanus; ^g^other diseases; ^h^congenital anomaly; ^i^diarrhea.

We estimated the number of cause-specific child deaths averted by comparing the number of cause-specific child deaths published and the estimates assuming that child mortality remain the same as in the year 2000 on a yearly basis.
